# Reporting of harms in systematic reviews focused on naltrexone: a cross-sectional study

**DOI:** 10.3389/fpsyt.2025.1597019

**Published:** 2025-09-22

**Authors:** Joseph Schnitker, Lindsey Purcell, Morgan Garrett, Holly Flores, Audrey Wise, Micah Kee, Brayden Rucker, Adam Khan, Jason Beaman, Matt Vassar

**Affiliations:** ^1^ Office of Medical Student Research, Oklahoma State University Center for Health Sciences, Tulsa, OK, United States; ^2^ Department of Surgery, University of Kansas Medical Center, Kansas City, KS, United States; ^3^ Department of Obstetrics, Gynecology and Reproductive Sciences, UTHealth Houston, Houston, TX, United States; ^4^ Department of Obstetrics and Gynecology, Baylor Scott and White Medical Center, Temple, TX, United States; ^5^ Department of Internal Medicine, Oklahoma State University Medical Center, Tulsa, OK, United States; ^6^ Department of Anesthesiology, University of Oklahoma College of Medicine, Oklahoma City, OK, United States; ^7^ Department of Psychiatry and Behavioral Sciences, Oklahoma State University Center for Health Sciences, Tulsa, OK, United States

**Keywords:** naltrexone, systematic reviews, harms reporting, AMSTAR-2, PRISMA harms, adverse effects, cross-sectional analysis

## Abstract

**Background:**

Naltrexone is a pharmacological intervention widely used for alcohol use disorder (AUD), opioid use disorder (OUD), and several off-label conditions. Systematic reviews (SRs) play a critical role in synthesizing data on the efficacy and safety of such interventions to inform clinical guidelines and decision-making. However, adequate reporting of harms in SRs remains inconsistent, limiting the ability to fully assess the safety profile of naltrexone. This study evaluates completeness of harms reporting and methodological quality in SRs focusing on naltrexone.

**Methods:**

A comprehensive search of MEDLINE, EMBASE, Epistemonikos, and the Cochrane Database of Systematic Reviews was conducted. The study employed masked, duplicate screening and data extraction. Included SRs were evaluated for completeness of harms reporting using the Preferred Reporting Items for Systematic Reviews and Meta-Analyses (PRISMA) harms checklist and other established frameworks. Methodological quality was appraised using the A MeaSurement Tool to Assess Systematic Reviews-2 (AMSTAR-2) tool, and primary study overlap among SRs was assessed through corrected covered area (CCA) analysis.

**Results:**

A total of 87 SRs were included in the analysis. Only 1.1% (1/87) utilized severity scales to classify harms, and 4.6% (4/87) defined harms in their methods. Nearly half (48.3%) of SRs failed to address harms as either a primary or secondary outcome. A total of 82.8% (72/87) of SRs were rated as “critically low” quality by AMSTAR-2. Statistical analysis revealed a significant relationship between “critically low” AMSTAR-2 ratings and incomplete harms reporting (*p* = 0.0486). Additionally, four SR pairs demonstrated “high” overlap (>50%) of primary studies, accompanied by inconsistencies in harms reporting.

**Conclusion:**

Our findings underscore the critical need for improved and standardized harms reporting in SRs on naltrexone. Inconsistent and incomplete reporting limits the ability of clinicians to fully assess the safety profile of naltrexone within systematic reviews. Adopting established frameworks such as PRISMA harms extensions and severity scales is imperative to enhance transparency and reliability in SRs. This study advocates for methodological improvements in SRs to support comprehensive safety evaluations and evidence-based prescribing of naltrexone.

## 1 Introduction

Harms reporting is crucial for interventions with rapidly expanding indications and recently updated literature. For example, naltrexone has been approved by the Food and Drug Administration (FDA) as an oral formulation for the treatment of alcohol use disorder (AUD) since 1984 and as an extended-release intramuscular injectable to treat both AUD and opioid use disorder (OUD) since 2006 (1, 2). Importantly, newer indications such as obesity and dermatologic conditions have been documented (3, 4). Given the growing list of possible indications for naltrexone therapy, medical literature, specifically systematic reviews (SRs), must provide a balanced reporting of benefits and harms, as SRs commonly underpin clinical practice guidelines, which guide clinical decision-making. Reporting complications of naltrexone is important for clinicians to adequately interpret the drug’s full safety profile. Furthermore, it has been documented that patients with higher levels of the urinary metabolite of naltrexone, 6-beta-naltrexol, experienced several side effects (including nausea, headache, anxiety, and erection), necessary information for physicians to consider when prescribing naltrexone (5).

SRs are the highest form of evidence offered within medical literature. However, SRs have demonstrated several inconsistencies, especially with regard to reporting outcomes data (6–8). Qureshi et al. also reported on such inconsistencies, finding that SRs often fail to capture the entirety of adverse events, such as rate, severity, and timing (9). Omitting results or failing to completely report information is critical, as harms data may allow readers to reach inaccurate conclusions that have downstream effects on clinical decision-making and, ultimately, patient care.

Systematic reviews have the unique ability to synthesize relevant studies on a particular topic and can draw timely and informative summary effects (10). They are often a reference source for physicians to ensure that their clinical decisions are high-quality and evidence-based (11–13). Several established reporting guidelines specifically address adverse effect reporting—Consolidated Standards of Reporting Trials (CONSORT) harms for randomized trials, Preferred Reporting Items for Systematic Reviews and Meta-Analyses (PRISMA) harms for systematic reviews, and the Cochrane Handbook chapter on adverse effects—which specify key items such as prespecifying adverse events, ascertainment methods, appropriate denominators, severity grading, and balanced presentation. However, adherence remains inconsistent (14, 36–39). To our knowledge, no studies thus far have analyzed the extent to which SRs on naltrexone address harms. Thus, we aim to 1) evaluate harms reporting in SRs on naltrexone, 2) determine if any relationships exist between completeness of harms reporting and study characteristics, and 3) evaluate the reporting of harms between SRs with common primary studies.

## 2 Methods

### 2.1 Study design

This cross-sectional analysis followed the PRISMA guidelines (15, 16). Our study was not subject to Institutional Review Board (IRB) approval, as it did not involve human subjects.

### 2.2 Harms terminology

**Figure 1:**
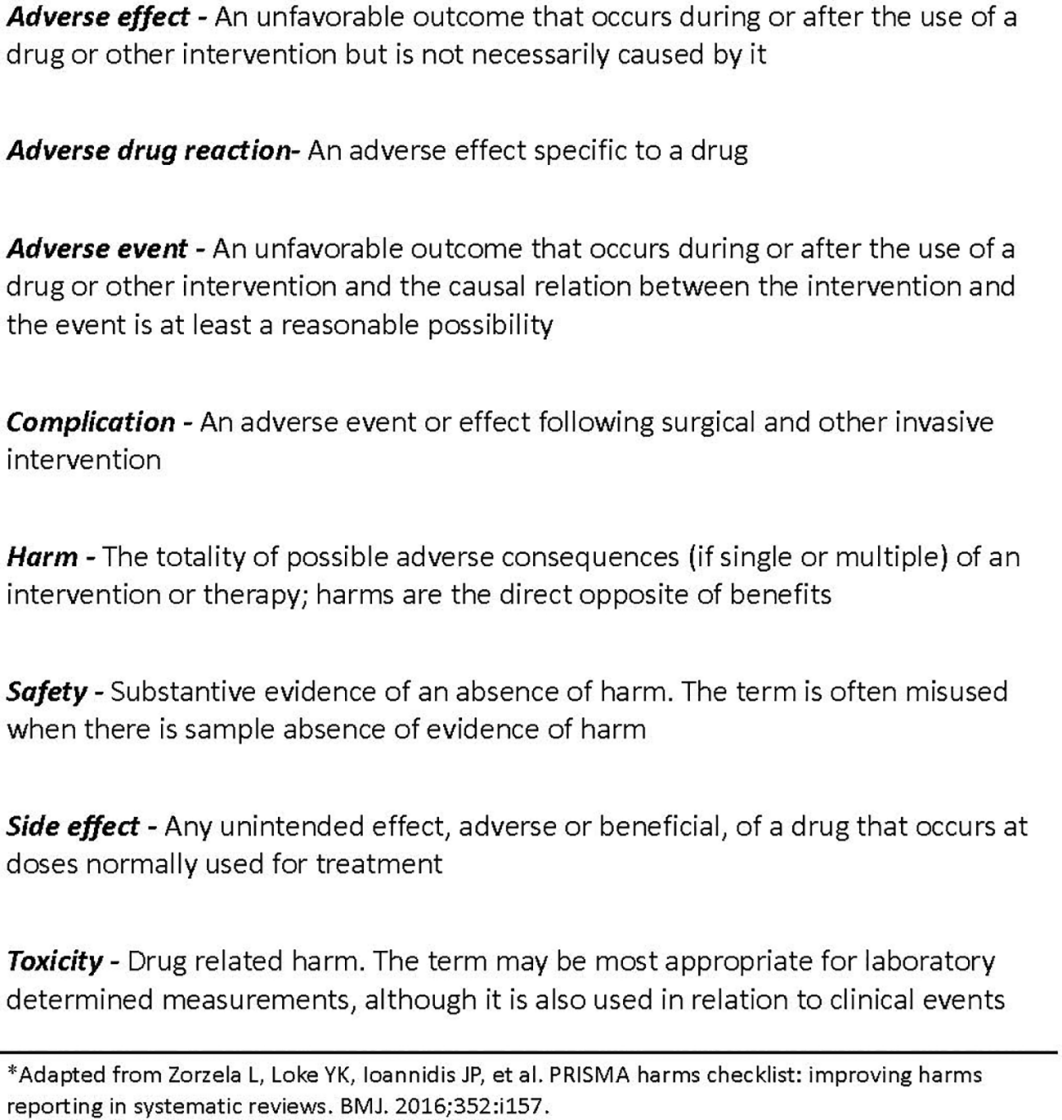
Glossary of terms*.

In accordance with the PRISMA harms group, we used terms and definitions for harms displayed in **Figure 1** (17).

### 2.3 Search strategy

An SR librarian developed a search string to search the databases MEDLINE (PubMed and Ovid), EMBASE, Epistemonikos, and Cochrane Database of Systematic Reviews. The strategies combined controlled vocabulary (e.g., MeSH *Naltrexone* in MEDLINE; Emtree *naltrexone* in EMBASE) with text words for the generic name, chemical synonyms (e.g., “naltrexone hydrochloride” and “N-cyclopropylmethylnoroxymorphone”), and brand names (e.g., ReVia, Vivitrol, Depade, Nodict, Trexan, and Vivitrex). Where available, we applied systematic review limits/filters (e.g., PubMed “Systematic Review” filter and database-specific SR limits). Afterward, we uploaded the records obtained to Rayyan (https://rayyan.qcri.org/), an SR screening platform. Two investigators (JS and LP) independently screened records in a masked, duplicate fashion for inclusion and removed all duplicates. Following title and abstract screening, investigators were unmasked, and any disagreements were resolved by a third-party investigator (MG).

### 2.4 Search string

The search string was uploaded to the Open Science Framework (OSF) (18).

### 2.5 Eligibility criteria

To be included in our sample, we required the following criteria: 1) the publication must be an SR regardless of having a meta-analysis or not, and 2) the SR must be designated to evaluate naltrexone for both FDA-approved uses (AUD and OUD) and off-label uses. Studies had to be in English and only include human subjects. Studies were excluded if they were not related to naltrexone or were not SRs.

### 2.6 Training

Two investigators (JS and LP) were trained on SRs via the Johns Hopkins Systematic Review course (19). Investigators were instructed on how to extract harms items from SRs in other fields of medicine using a pilot-tested Google Form. Training on A MeaSurement Tool to Assess Systematic Reviews-2 (AMSTAR-2) in video and lecture format was also provided. Data from the AMSTAR-2 tool were compiled and interpreted using a pilot-tested Google Form. Senior author MV—who has published a multitude of studies evaluating the methodology of SRs—led all training (20–23).

### 2.7 Data extraction

Two investigators (JS and LP) extracted study characteristics using a pilot-tested Google Form. The characteristics included title, journal, Rayyan ID, and nine variables to evaluate studies (e.g., whether harms were evaluated as an outcome and whether the SR mentioned adherence to PRISMA guidelines) (16). Using methods similar to those of Mahady and colleagues, the same investigators extracted the data items listed in **Table 1** from included SRs, coding each item as “yes” or “no” (24). Using methods similar to those of Qureshi and colleagues, they also extracted the items listed in **Table 2**, again coding “yes” or “no” unless free response or multiple choice was required (9, 25, 26). All extraction was performed independently in masked duplicates; disagreements were resolved by discussion, with MG adjudicating as needed.

**Table 1:** Mahady assessment for completion of harms reporting (n = 87).

Harms assessment	Frequency (%)	
	Yes	No
1. Are harms stated in the title or abstract?	32 (36.8)	55 (63.2)
2. Are harms presented in the introduction?	21 (24.1)	66 (75.9)
3. Are harms listed and separately defined in the methods?	4 (4.6)	83 (95.4)
4. Are grades and/or severity scales used to classify harms in the methods?	1 (1.1)	86 (98.9)
5. Is there a method of harms data collection stated in the methods?	35 (40.2)	52 (59.8)
6. Is there a planned statistical analysis for harms stated in the methods?	23 (26.4)	64 (73.6)
7. Is the number of patients available for harms analyses stated in the results?	25 (28.7)	62 (71.3)
8. Is the number of treatment discontinuations in each arm reported in the results?	13 (14.9)	74 (85.1)
9. Are absolute figures for each harm in treatment and control groups presented in the results?	15 (17.2)	72 (82.8)
10. Were limitations of harms analyses discussed?	11 (12.6)	76 (87.4)
11. Is a balanced discussion of harms and benefits provided?	34 (39.1)	53 (60.9)
12. Did the authors discuss what future research would be needed to better clarify harms?	17 (19.5)	70 (80.5)
Total systematic reviews		
Completed 0% of harms items	28 (32.2)	
Completed 1%–49.9% of harms items	42 (48.3)	
Completed 50% or more of items	17 (19.5)	

**Table 2:** Qureshi assessment for completion of harms reporting (n = 87).

Harms assessment	No. (%)
1. Did the study prespecify any harms?	
Yes	36 (41.4)
No	51 (58.6)
2a. What were the types of harms assessed?	Uploaded to OSF*
2b. What language was used to describe those types of harms?	Uploaded to OSF*
2c. What were the effect estimates used to assess harms?	
Mean difference	3 (3.4)
Odds ratio	7 (8.0)
Relative risk	1 (1.1)
Risk difference	3 (3.4)
Risk ratio	10 (11.5)
None	29 (33.3)
Not applicable	34 (39.1)
3. Was a prespecified protocol available that addressed harms?	
Yes	18 (20.7)
No	4 (4.6)
Could not find protocol	56 (64.4)
Available protocol did not address harms	9 (10.3)
4. Were any specific harms or harms language included in the search strategy?	
Yes	5 (5.7)
No	82 (94.3)
5. Was a given harm assessed qualitatively or quantitatively (i.e., within a meta-analysis)?	
Both quantitative and qualitative	2 (2.3)
Only quantitative	20 (23.0)
None	64 (73.6)
Not applicable	1 (1.1)
6. If a given harm was assessed quantitatively, what models and assumptions were used?	
Fixed effects	3 (3.4)
Random effects	12 (13.8)
Fixed and random effects	7 (8.0)
Not applicable	65 (74.7)
7. Did the authors apply selection criteria to reported harms?	
Yes	4 (4.6)
No	83 (95.4)

*OSF, Open Science Framework.

To quantify how much the included SRs relied on the same primary studies, we calculated the corrected covered area (CCA), which standardizes overlap by accounting for both the number of SRs and the number of unique studies (27). We first constructed a citation matrix listing all included SRs (columns) against all primary studies (rows), marking presence/absence. We then computed CCA = (C − U)/[(U × R) − U], where C is the total number of primary study citations across all SRs (sum of matrix entries), U is the number of unique primary studies, and R is the number of SRs. Higher CCA indicates greater redundancy of evidence across reviews. Following published guidance, we interpreted overlap as minimal (<20%), moderate (20%–50%), or high (>50%). For pairs of SRs with ≥50% overlap (high), we performed targeted, side-by-side comparisons (“dyads”) of harms reporting to evaluate consistency (e.g., whether similar adverse events, definitions, and severities were presented despite drawing on largely the same primary evidence).

The authors performed a quality appraisal of each SR using the AMSTAR-2 instrument (28). Each of the 16 items was scored as “yes”, “partial yes”, or “no” depending on whether all criteria were met, some criteria were met, or the criteria were insufficiently met to warrant “yes” or “partial yes”. Items 11, 12, and 15 pertain to SRs with a meta-analysis; reviews without a meta-analysis were therefore scored out of 13 rather than 16. AMSTAR-2 assigns overall confidence ratings based on the presence of critical and non-critical flaws: reviews with no or only one non-critical weakness were rated *high*, those with more than one non-critical weakness but no critical flaws were rated *moderate*, those with one critical flaw (with or without non-critical weaknesses) were rated *low*, and those with more than one critical flaw (with or without non-critical weaknesses) were rated *critically low*. Using these criteria, each SR in our sample was classified into a quality category using the AMSTAR-2 quality assessment generator.

### 2.8 Data analysis

The characteristics of included studies, harms data, and AMSTAR-2 data for all included SRs were reported in frequency and percentage. A bivariate analysis was performed to determine if any associations existed between quality rating, general characteristics, and harms reporting. The nature of the data (i.e., statistical assumptions and distributional qualities) influenced the choice of statistical test. A *p*-value less than or equal to 0.05 was considered significant. For the CCA, the following were reported: the number of primary studies across all SRs, the range of primary studies used by an included SR, and the number of primary studies reported in one, two or more, and five or more included SRs (26). Overall, CCA was calculated across all SRs. Lastly, in all pairs of SRs with a high overlap of primary studies, individual harms and reporting items were compared (27). Stata 16.1 (StataCorp, LLC, College Station, TX) was used for data analysis. Data scrubbing was conducted using Microsoft Excel.

### 2.9 Reproducibility

To maximize transparency and reproducibility, all study materials were publicly archived on the OSF (https://osf.io/zae45/) (18). The repository includes the full protocol with prespecified objectives, eligibility criteria, outcomes, and analysis plans; complete database search strategies; the deidentified, raw screening and extraction datasets; the pilot-tested extraction forms used by investigators; and the statistical code used for the corrected covered area analysis. Screening and data extraction were conducted independently in masked duplicates, with disagreements resolved by consensus or third-party adjudication; AMSTAR-2 assessments followed the same process. Version history is preserved in the OSF to document any updates to methods or data, and all materials are available to enable verification, replication, and extension of our analyses.

## 3 Results

### 3.1 Screening process

**Figure 2:**
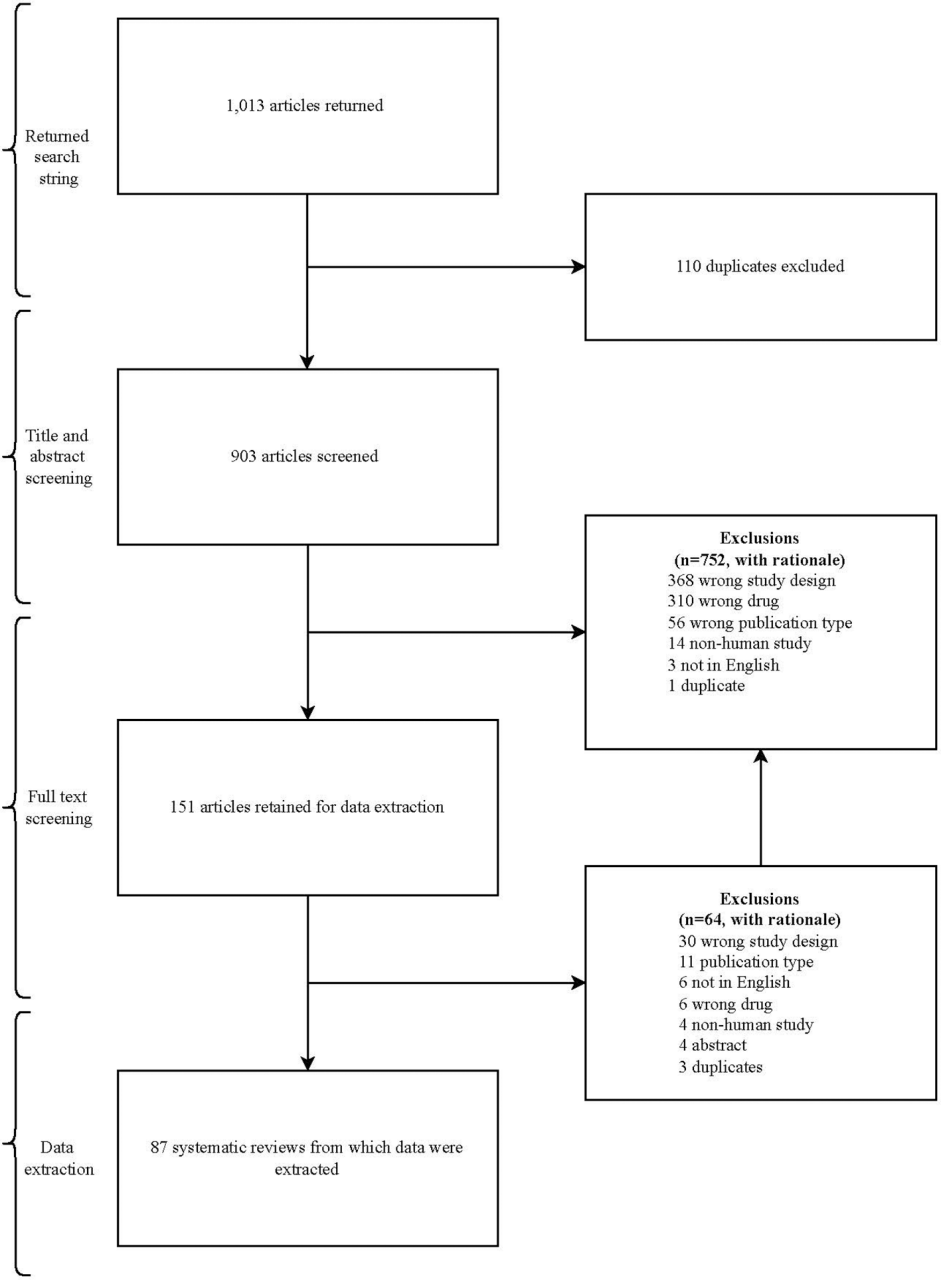
Flow diagram of study selection.

Our search returned 1,013 articles. After duplicates were removed, 903 articles were eligible for title and abstract screening. An additional 752 articles were excluded, leaving 151 articles eligible for full-text review. The reasons for exclusion in each phase of the screening process are presented in **Figure 2**.

### 3.2 Characteristics of included studies

A total of 87 SRs were included. Of the 87 SRs, 44 (44/87, 50.6%) reported adherence to PRISMA, 56 (56/87, 64.4%) found naltrexone as a favorable intervention, and 37 (37/87, 42.5%) did not report a funding source. Additionally, 18 SRs (18/87, 20.7%) reported harms as a primary outcome, 27 (27/87, 31.0%) reported harms as a secondary outcome, and 42 (42/87, 48.3%) did not report harms as a primary or secondary outcome. The general characteristics of included SRs can be found in **Table 3**.

**Table 3:** Summary of characteristics of included studies (n = 87).

Review characteristics	No. (%)
Indications	
Alcohol use disorder	27 (31.0)
Opioid use disorder	14 (16.1)
Obesity	8 (9.2)
Cholestatic pruritus	4 (4.6)
Smoking	4 (4.6)
Stimulant use disorder	4 (4.6)
Opioid-induced constipation	3 (3.4)
Behavioral addictions	2 (2.3)
Chronic pain	2 (2.3)
Schizophrenia	2 (2.3)
Trichotillomania	2 (2.3)
Autism	1 (1.1)
Borderline personality disorder	1 (1.1)
Chronic kidney disease-associated pruritus	1 (1.1)
Crohn’s disease	1 (1.1)
Deliberate foreign body ingestion	1 (1.1)
Dissociative disorders	1 (1.1)
Eating disorders	1 (1.1)
Non-cancer pain management	1 (1.1)
Non-suicidal self-injury	1 (1.1)
Obstructive sleep apnea	1 (1.1)
Opioid-induced pruritus	1 (1.1)
Polydrug dependence	1 (1.1)
Prader–Willi syndrome	1 (1.1)
Tardive dyskinesia	1 (1.1)
Uremic pruritus	1 (1.1)
Study mentions adherence to PRISMA^a^	
Yes	44 (50.6)
No	43 (49.4)
Intervention favorable	
Yes	56 (64.4)
No	31 (35.6)
Was harm a primary or secondary outcome, or neither?	
Primary outcome	18 (20.7)
Secondary outcome	27 (31.0)
Neither	42 (48.3)
Conflicts of interest	
Yes	27 (31.0)
No	41 (47.1)
Not stated	19 (21.8)
Funding source	
Not funded	14 (16.1)
Not mentioned	37 (42.5)
Private	5 (5.7)
Public	31 (35.6)
AMSTAR-2 rating^b^	
High	2 (2.3)
Moderate	1 (1.2)
Low	12 (13.8)
Critically low	72 (82.8)

a Preferred Reporting Items for Systematic Reviews and Meta-Analyses.

b A MeaSurement Tool to Assess Systematic Reviews.

### 3.3 Harms extraction

Of the 87 SRs in our analysis, one SR (1/87, 1.1%) classified grades/severity scales for harms in the methods, and four SRs (4/87, 4.6%) listed and separately defined harms in the methods. We found that 11 SRs (11/87, 12.6%) of the included studies discussed limitations to assessing harms. Five SRs (5/87, 5.7%) included harms language in their search strategies, 18 SRs (18/87, 20.7%) followed a protocol that addressed harms, and 36 (36/87, 41.4%) prespecified harms. A total of 17 SRs completed 50% or more of harms items (17/87, 19.5%). A comprehensive list of evaluated harms items can be found in **Tables 1** and **2**.

### 3.4 Corrected covered area

Of our 87 included SRs, our CCA analysis included primary studies from 85 SRs. In total, 2,475 primary studies were cited. The total number of unique primary studies included across all SRs was 1,791. The fewest number of primary studies cited by an SR was 2, and the most was 151. Of our 85 included SRs for CCA analysis, there were 1,463 primary studies cited once. There were 284 primary studies cited in two to four SRs and 44 primary studies cited in five or more SRs. For the eligible 85 SRs, the overall CCA was 0.45%. Four dyads were considered “high” overlap, 35 dyads were considered “moderate” overlap, and the remaining dyads were considered “minimal” overlap. The results of CCA are found in **Table 4**.

**Table 4:** Naltrexone harms reported by the paired reviews with a corrected covered area (CCA) >50% (n = 4 pairs of reviews).

Harms reported	
Dyad 411 (75% overlap)	
Kirchmayer et al., 2001	Kirchmayer et al, 2003
Side effects	Side effects
Adverse effects	
Percent of harms also included in Kirchmayer et al., 2003	Percent of harms also included in Kirchmayer et al., 2001
1/2 (50.0%)	1/1 (100.0%)
Dyad 493 (50% overlap)	
Kirchmayer et al., 2003	Minozzi et al., 2006
Side effects	Side effects
Percent of harms also included in Minozzi et al., 2006	Percent of harms also included in Kirchmayer et al., 2003
1/1 (100.0%)	1/1 (100.0%)
Dyad 946 (50% overlap)	
Pettinati et al., 2006	Rosner et al., 2010
Side effects	Side effects
Nausea	Nausea
Vomiting	Vomiting
Depression	Depression
Low energy	Stomach pain
Anxiety	Loss of appetite
Headache	Daytime drowsiness
Rash	Nightmares
Decreased alertness	Fatigue
	Insomnia
	Lethargy
	Weakness
	Somnolence
	Blurred vision
	Decreased libido
	Withdraw due to side effects
	Dizziness
Percent of harms also included in Rosner et al., 2010	Percent of harms also included in Pettinati et al., 2006
4/9 (44.4%)	4/17 (23.5%)
Dyad 2421 (73% overlap)	
Khera et al., 2016	Singh and Singh 2019
Adverse events	Adverse events
Discontinuation due to adverse events	Nausea
	Vomiting
	Constipation
	Diarrhea
	Dry mouth
	Dizziness
	Increased systolic blood pressure
	Increased heart rate
	Depression
	Suicidal ideation
	Seizure
	Exacerbation of angle closure glaucoma
	hepatic dysfunction
	Insomnia
Percent of harms also included in Singh and Singh 2019	Percent of harms also included in Khera et al., 2016
1/2 (50.0%)	1/15 (6.7%)

### 3.5 AMSTAR-2 assessment

Of the 87 included SRs, two SRs (2/87, 2.3%) were graded as “high” quality, one SR (1/87, 1.1%) was graded as “moderate” quality, 12 SRs (12/87, 13.8%) were graded as “low” quality, and 72 (72/87, 82.8%) were graded as “critically low” quality (**Table 3**).

### 3.6 Associations

A Kruskal–Wallis test showed a significant relationship between studies graded “critically low” via AMSTAR-2 and completeness of harms reporting (*p* = 0.0486). Also, a significant relationship was found between studies that specified harms as an outcome and completeness of harms reporting (*p* = 0.0001). No significant association was determined between completeness of harms reporting and whether the SR reported adherence to PRISMA.

## 4 Discussion

We observed a lack of harms reporting in SRs concerning naltrexone—19.5% of our included SRs reported on half or more of the assessed harms items, and 28 SRs made no mention of harms (24). Most SRs in our sample failed to address harms within the methodology, specifically in regard to classifying and listing harms. Of concern, only one SR used a grade or severity scale for classifying harms. Our findings suggest that harms reporting is scarce, and improvements are needed to provide clinicians with accurate and complete safety profiles regarding naltrexone. Here, we discuss our findings along with relevant studies, give examples of underreported harms items as well as their implications, and provide recommendations to improve reporting.

In accordance with our findings, studies have previously shown that harms reporting is deficient in SRs. For example, Papanikolaou and Ioannidis conducted a study examining SRs published in the Cochrane Database and found that of the 138 SRs with at least 4,000 subjects, 77 SRs reported no harms data (29). Furthermore, the authors found that when harms reporting was deficient in a given SR, specific harms were presented adequately in 29% of the primary studies, suggesting that failure to report harms took place not only at the SR level (29). Additionally, Mahady and their colleagues looked at 78 gastroenterology SRs and found that one-third of the included SRs did not address harms at all and that the number of figures on harms was lacking, especially compared to the number of figures on efficacy (24). The results of these studies, along with ours, suggest that underreporting of harms is prevalent.

In our CCA analysis, we found that many of our included SRs cited the same primary studies. For example, Dyad 2421 shared 73% of the cited primary studies. However, harms reporting was very different. This dyad discussed adverse events and discontinuations due to adverse events in one SR, while the other SR discussed adverse events, such as nausea, vomiting, constipation, diarrhea, dry mouth, dizziness, increased blood pressure and heart rate, depression, suicidal ideation, seizure, exacerbation of angle closure glaucoma, hepatic dysfunction, and insomnia. This suggests the possibility of reporting bias of harms among SRs concerning naltrexone and that improvements in harms reporting in SRs are needed to reduce such inconsistency.

In our study, almost all SRs failed to use grades or severity scales to classify harms. This finding is not benign and may have multiple downstream effects. The Substance Abuse and Mental Health Services Administration (SAMHSA) reports “common” side effects (nausea, headache, etc.) and “serious” side effects (pain, tissue death requiring surgery, etc.) of naltrexone (30). Interestingly, “serious” side effects are not defined. Thus, clinicians and researchers are left to speculate on the true severity of a “serious” side effect. To mitigate this uncertainty, other studies have applied severity scales to classify and define harms. For example, a study evaluating brodalumab for the treatment of psoriasis used the Columbia-Suicide Severity Rating Scale (C-SSRS) to determine if suicidal ideations and behaviors were related to initiating pharmacotherapy (31). By reviewing results provided by this scale, the authors were able to conclude that suicidal ideations and behaviors were likely unrelated to brodalumab. We argue that the implementation of standardized scales is crucial to SRs owing to the ease of data synthesis when combining similar harms from primary studies. Moreover, the use of severity scales allows SR authors to provide a meaningful discussion on harms along with the translation of harms into clinical decision-making.

Furthermore, the classification of harms provides clinicians with additional information when determining the best plan of care for a patient. For example, one side effect of naltrexone classified as “serious” is a depressed mood. This particular harm poses unique challenges to clinicians, as depressed mood may be a side effect of treatment or related to a given diagnosis. Linden expanded on these challenges by stating that within the field of psychiatry, there is inherent difficulty in differentiating side effects, as they may be attributable to patient behavior. Linden also described the use of a checklist Unwanted Event–Adverse Treatment Reaction checklist (UE-ATR) to record, monitor, and classify adverse events related to psychotherapy (32). Applying a similar checklist to pharmacological therapy may encourage clinicians to account for harms with great accuracy and allow for a standard comparison of harms. Use of a standardized checklist would likely reduce the burden of characterizing ambiguous harms, especially in higher-complexity cases that require multiple therapies.

### 4.1 Recommendations

Because our study found deficiencies in harms reporting on naltrexone, we first recommend an overall improvement in harms reporting. This could be attained by adherence to standardized methods of harms reporting, such as PRISMA and CONSORT harms extensions (17, 33). Second, we suggest improvements be made to SRs using grades or severity scales when reporting harms to reduce ambiguity. Petrova et al. and Koh et al. discussed potential methods and tools for reporting the severity of harms of medical interventions (34, 35).

Furthermore, to reduce ambiguity and improve comparability, systematic reviews could prespecify and apply standardized grading frameworks for adverse events [e.g., map events to Common Terminology Criteria for Adverse Events (CTCAE) (5-point grades) and use the Naranjo Algorithm for causality when attribution is unclear], classify suicidal ideation/behavior using C-SSRS, and use systems such as ABACUS for general drug reaction classification (40–42). Practically, protocols could name target scales *a priori*; define how non-standard labels (e.g., “serious”, “severe”, and “clinically significant”) will be mapped to scale grades, extract, and report both counts and grade distributions (e.g., Grade ≥3); and use consistent denominators and exposure windows for grade-stratified summaries. Applying these frameworks standardizes terminology, clarifies thresholds for seriousness, and supports meta-analysis where appropriate, thereby improving clarity, reproducibility, and clinical interpretability of harms reporting. Notably, 82.7% of SRs in our sample were rated “critically low” using AMSTAR-2, underscoring the need for better methods; until harms reporting improves, clinicians should exercise caution with naltrexone and monitor patients closely.

To operationalize these recommendations, future SRs could register a protocol that prespecifies adverse-event definitions/lists, ascertainment windows, severity grading (with protocol-listed scales and explicit mapping rules for non-standard terms), denominators/time-at-risk, and rules for zero-event data to reduce selective reporting and clarify rate calculations; expand information sources beyond trials to include long-term extensions, observational cohorts/registries, and post-marketing surveillance to better capture long-latency or infrequently collected harms; use dual, standardized extraction that records adverse event (AE) definition, assessment method, grade, timing window, denominator, exposure duration, and whether the AE was prespecified to increase accuracy and comparability; synthesize using both counts and rates and, for rare events, prespecify effect measures and sensitivity analyses or provide a structured narrative when meta-analysis is inappropriate to yield stable, transparent estimates; and report according to PRISMA harms with balanced presentation and public sharing of extraction sheets/code to strengthen transparency and reproducibility. Collectively, these steps improve capture of long-latency or infrequently collected harms, increase completeness and comparability, and enhance interpretability and reproducibility for clinicians.

### 4.2 Strengths and limitations

Addressing study strengths, we executed a study design created specifically for transparency and reproducibility. Documenting our strategies prior to starting the project, we uploaded a detailed protocol to the OSF for reference (18). We routinely uploaded any changes, updates, or modifications. Additionally, we worked with an SR librarian to develop a search strategy including numerous bibliographic databases responsible for routinely cataloging reviews. Screening for harms and AMSTAR-2 in a masked, duplicate fashion allowed the authors to extract accurate data. While there were many strengths within our study, some limitations are noted.

Our analyses are limited to harms collected and reported in the included trials and SRs; because randomized trials often have restricted eligibility and short follow-up, long-term or infrequently captured adverse events may be underrepresented, and complementary sources (e.g., long-term extensions, observational cohorts, registries, and post-marketing surveillance) may be required to detect them. Unclear or unreported items were coded as *not reported* per prespecified rules (no imputation), which likely biases completeness estimates downward and may amplify between-review differences. Although extraction and AMSTAR-2 ratings were performed in masked duplicates with adjudication, some judgments remain partially subjective. Two SRs lacked full primary study lists and were excluded from the CCA, which could modestly affect overlap estimates and dyad composition. Generalizability is limited due to the cross-sectional nature of our study. Additionally, the quality assessment used AMSTAR-2, a checklist developed in 2012; therefore, studies published prior to this could not follow this set of guidelines.

## 5 Conclusion

Our analysis found the harms of naltrexone to be underreported in SRs. Considering the important role of SRs in medicine, harms should be well-reported. Standardized reporting methods currently exist that could improve harms reporting, but adherence to them is lacking. The benefits and harms of naltrexone should influence clinical decision-making when using the medication. However, until harms reporting is more complete, including defined grades/severity scales, properly informed decisions on the use of naltrexone are deficient.

## Data Availability

The original contributions presented in the study are included in the article/**Supplementary Material**. Further inquiries can be directed to the corresponding author.
