# Supplementary material for: Reporting of harms in systematic reviews focused on naltrexone: a cross-sectional study
Source: Front Psychiatry. 2025 Sep 22;16:1597019. doi: 10.3389/fpsyt.2025.1597019 (PMC12497844; doi:10.3389/fpsyt.2025.1597019)
Supplement: Supplementary file 1 [file DataSheet1.pdf]

Naltrexone

Pubmed

(((("Naltrexone"[Mesh]) AND (systematicreview[Filter])) OR ((ReVia OR Vivitrol OR Depade OR "N-Cyclopropylmethylnoroxymorphone" OR "naltrexone hydrochloride" OR Adepend OR Antaxone OR Celupan OR Depade OR Nalorex OR Narcoral OR Nemexin OR Nodict OR Trexan OR Vivitrex) AND (systematicreview[Filter]))) AND (systematicreview[Filter]))

EMBASE

#18	#17 AND 'systematic review'/de	473
#17	#1 OR #2 OR #3 OR #4 OR #5 OR #6 OR #7 OR #8 OR #9 OR #10 OR #11 OR #12 OR #13 OR #14 OR #15 OR #16	15,986*
#16	trexan	101*
#15	vivitrex	22*
#14	nodict	1*
#13	nemexin	48*
#12	narcoral	1*
#11	nalorex	58*
#10	depade	18*
#9	celupan	4*
#8	antaxone	28*
#7	adepend	3*
#6	'naltrexone hydrochloride'	223*
#5	'n cyclopropylmethylnoroxymorphone'	0*
#4	depade	18*
#3	vivitrol	323*
#2	revia	315*
#1	'naltrexone'/exp	15,923*

Ovid MEDLINE

1 exp Naltrexone/ or Naltrexone.mp. 10854
2 ReVia.mp. [mp=title, abstract, original title, name of substance word, subject heading word, floating sub-heading word, keyword heading word, organism supplementary concept

word, protocol supplementary concept word, rare disease supplementary concept word, unique identifier, synonyms] 37
3 Vivitrol.mp. [mp=title, abstract, original title, name of substance word, subject heading word, floating sub-heading word, keyword heading word, organism supplementary concept word, protocol supplementary concept word, rare disease supplementary concept word, unique identifier, synonyms] 74
4 Depade.mp. [mp=title, abstract, original title, name of substance word, subject heading word, floating sub-heading word, keyword heading word, organism supplementary concept word, protocol supplementary concept word, rare disease supplementary concept word, unique identifier, synonyms] 1
5 N-Cyclopropylmethylnoroxymorphone.mp. [mp=title, abstract, original title, name of substance word, subject heading word, floating sub-heading word, keyword heading word, organism supplementary concept word, protocol supplementary concept word, rare disease supplementary concept word, unique identifier, synonyms] 1
6 "naltrexone hydrochloride".mp. [mp=title, abstract, original title, name of substance word, subject heading word, floating sub-heading word, keyword heading word, organism supplementary concept word, protocol supplementary concept word, rare disease supplementary concept word, unique identifier, synonyms] 166
7 Adepend.mp. [mp=title, abstract, original title, name of substance word, subject heading word, floating sub-heading word, keyword heading word, organism supplementary concept word, protocol supplementary concept word, rare disease supplementary concept word, unique identifier, synonyms] 0
8 Antaxone.mp. [mp=title, abstract, original title, name of substance word, subject heading word, floating sub-heading word, keyword heading word, organism supplementary concept word, protocol supplementary concept word, rare disease supplementary concept word, unique identifier, synonyms] 2
9 celupan.mp. [mp=title, abstract, original title, name of substance word, subject heading word, floating sub-heading word, keyword heading word, organism supplementary concept word, protocol supplementary concept word, rare disease supplementary concept word, unique identifier, synonyms] 1
10 Depade.mp. [mp=title, abstract, original title, name of substance word, subject heading word, floating sub-heading word, keyword heading word, organism supplementary concept word, protocol supplementary concept word, rare disease supplementary concept word, unique identifier, synonyms] 1
11 Nalorex.mp. [mp=title, abstract, original title, name of substance word, subject heading word, floating sub-heading word, keyword heading word, organism supplementary concept word, protocol supplementary concept word, rare disease supplementary concept word, unique identifier, synonyms] 1
12 Narcoral.mp. [mp=title, abstract, original title, name of substance word, subject heading word, floating sub-heading word, keyword heading word, organism supplementary concept

word, protocol supplementary concept word, rare disease supplementary concept word, unique identifier, synonyms] 0
13 Nemexin.mp. [mp=title, abstract, original title, name of substance word, subject heading word, floating sub-heading word, keyword heading word, organism supplementary concept word, protocol supplementary concept word, rare disease supplementary concept word, unique identifier, synonyms] 4
14 Nodict.mp. [mp=title, abstract, original title, name of substance word, subject heading word, floating sub-heading word, keyword heading word, organism supplementary concept word, protocol supplementary concept word, rare disease supplementary concept word, unique identifier, synonyms] 0
15 Vivitrex.mp. [mp=title, abstract, original title, name of substance word, subject heading word, floating sub-heading word, keyword heading word, organism supplementary concept word, protocol supplementary concept word, rare disease supplementary concept word, unique identifier, synonyms] 7
16 trexan.mp. [mp=title, abstract, original title, name of substance word, subject heading word, floating sub-heading word, keyword heading word, organism supplementary concept word, protocol supplementary concept word, rare disease supplementary concept word, unique identifier, synonyms] 10
17 1 or 2 or 3 or 4 or 5 or 6 or 7 or 8 or 9 or 10 or 11 or 12 or 13 or 14 or 15 or 16 10858
18 limit 17 to systematic reviews 187

Epistemonikos

naltrexone OR ReVia OR Vivitrol OR Depade OR N-Cyclopropylmethylnoroxymorphone OR naltrexone hydrochloride OR Adepend OR Antaxone OR Celupan OR Depade OR Nalorex OR Narcoral OR Nemexin, Nodict OR Trexan OR Vivitrex

Cochrane

ID	Search	Hits
#1	MeSH descriptor: [Naltrexone] explode all trees	1402
#2	Vivitrex	4
#3	Trexan	12
#4	Nalorex	3
#5	Narcoral	0
#6	Nemexin	5
#7	Nodict	1

#8	Depade	2
#9	naltrexone hydrochloride	159
#10	Adepend	1
#11	Celupan	2
#12	Antaxone	2
#13	ReVia	34
#14	Vivitrol	71
#15	Depade	2
#16	N-Cyclopropylmethylnoroxymorphone	0
#17	#1 OR #2 OR #3 OR #4 OR #5 OR #6 OR #7 OR #8 OR #9 OR #10 OR #11 OR #12 OR #13 OR #14 OR #15 OR #16	1519
	Systematic review	31
